# SiC Nanofibers as Long-Life Lithium-Ion Battery Anode Materials

**DOI:** 10.3389/fchem.2018.00166

**Published:** 2018-05-14

**Authors:** Xuejiao Sun, Changzhen Shao, Feng Zhang, Yi Li, Qi-Hui Wu, Yonggang Yang

**Affiliations:** ^1^Department of Materials Chemistry, School of Chemical Engineering and Materials Science, Quanzhou Normal University, Quanzhou, China; ^2^Jiangsu Key Laboratory of Advanced Functional Polymer Designand Application, Department of Polymer Science and Engineering, College of Chemistry, Chemical Engineering and Materials Science, Soochow University, Suzhou, China

**Keywords:** nanofibers, silicon carbide, anode, lithium-ion batteries, long-life

## Abstract

The development of high energy lithium-ion batteries (LIBs) has spurred the designing and production of novel anode materials to substitute currently commercial using graphitic materials. Herein, twisted SiC nanofibers toward LIBs anode materials, containing 92.5 wt% cubic β-SiC and 7.5 wt% amorphous C, were successfully synthesized from resin-silica composites. The electrochemical measurements showed that the SiC-based electrode delivered a stable reversible capacity of 254.5 mAh g^−1^ after 250 cycles at a current density of 0.1 A g^−1^. It is interesting that a high discharge capacity of 540.1 mAh g^−1^ was achieved after 500 cycles at an even higher current density of 0.3 A g^−1^, which is higher than the theoretical capacity of graphite. The results imply that SiC nanomaterials are potential anode candidate for LIBs with high stability due to their high structure stability as supported with the transmission electron microscopy images.

## Introduction

In recent years, due to the wide applications of lithium ion batteries (LIBs) in the portable electronic devices and especially in electric vehicles (EVs), higher criterion on their energy capacity and density as well as cycleability would be established. To further enhance the electrochemical performances of LIBs, it is necessary to find alternative electrode materials to replace the currently commercial ones e.g. graphitic carbons (An et al., 2017a,b; Zhang et al., 2018). SiC, a high bandgap semiconductor, is generally thought to be electrochemically inactive to Li, and often acts as backbone or buffer matrix to enhance the strength of electronic composite materials (Timmons et al., 2007; Jeon and Lee, 2014; Wang C. et al., 2015; Wang W. et al., 2015). But it is interesting that some researchers have studied the cycling performance of SiC-based nanocomposites as anode materials for LIBs. For example, nanoscopic carbon-coated SiC has been synthesized using a simple CVD method (Sri Devi Kumari et al., 2013), which delivered a reversible lithium storage capacity of about 1,200 mAh g^−1^ after 200 cycles, showing excellent cyclability. Zhang and Xu (2014) prepared a nanocrystalline SiC thin film electrode grown on the stainless steel, which showed a stable discharge capacity of 309 mAh g^−1^ over 60 cycles. Hu et al. (2016) produced SiC nanowires on the graphite paper, which delivered 397 mAh g^−1^ after 100 cycles. These results indicated that when a non-conducting SiC material reduces to nano size, it can also serve as anode materials for LIBs. As explained by Li et al. (2016), the capacity of SiC nanomaterials could be activated upon an intimate contact of SiC with graphite, which facilitates the electronic and ionic transport while suppressing the oxidation of SiC. Moreover, during the electrochemical studies of the SiC nanomaterials, the gradual enhancement of capacities was found, which has not been well studied. Up to date, the reports on SiC as anode material for LIBs are few. As a potential material for long-life LIBs, the lithium ions diffusion/reaction mechanism in/with SiC nanostructures needs to be further investigated, which would benefit to later similar studies.

SiC nanostructures can be synthesized by converting well defined SiO_2_ nanoarchitectures through carbothermal reduction at a high temperature over 1,400°C, by which the SiC products could preserve the structure regularity of the starting SiO_2_ nanomaterials (Miller et al., 1979; Martin et al., 1998). Nano-sized SiC materials can also be fabricated by pyrolysis of Si-containing polymers such as polybissilsesquioxanes (Wang C. et al., 2015; Zhang et al., 2015). In our previous work, we reported SiC/C composite nanotubes (69 wt% SiC and 31 wt% C) prepared via pyrolysis of resorcinol-formaldehyde (RF) resin-silica composite nanotubes at 1,400°C under argon atmosphere. The RF resin-silica composite was synthesized using a sol-gel transcription method taking a chiral gelator as template (Shao et al., 2017b). Herein, SiC nanofibers (with 7.5 wt% residual C) were fabricated using RF resin-silica composite nanofibers as the starting material and then applied as electrode materials for LIBs. The electrochemical data showed that the SiC-based electrode possessed superior lithium ion storage capability and cycling stability. Further, the electrochemical reaction mechanism of lithium and SiC nanofibers was proposed.

## Experimental

### General methods

Field emission scanning electron microscopy (FE-SEM, 4800 instrument) and transmission electron microscopy (TEM, FEI TecnaiG220) images were taken at 3.0 and 200 kV to observe the samples' morphologies and nanostructures, respectively. A Jobin Yvon Horiba HR 800 LabRAM confocal microprobe Raman system was applied to collect the Raman spectrum with Ar laser excitation at 514.5 nm and a power of 10 mW. Wide angle X-ray diffraction (WAXRD) patterns were obtained based on an X'Pert-Pro MPD X-ray diffractometer with Cu Kα radiation (1.542Å). Specific surface area and pore-size distribution were determined according to N_2_ adsorption isotherm measured from a Micromeritics Tristar II 3020 instrument via the Brunauer-Emmett-Teller (BET) and Barrett-Joyner-Halon (BJH) methods.

### Synthetic procedure of RF resin-silica composite and SiC nanofibers

Gelator D-1, whose molecular structure is shown in Figure S1, was synthesized according to the literature (Li et al., 2013; Zhang and Xu, 2014). A typical synthesis route of the resin-silica composite was as following: D-1 (200 mg, 0.31 mmol) and resorcinol (180 mg, 1.63 mmol) were dissolved in deionized water (35 mL) and ethanol (5.0 mL) mixed solution at 40°C under vigorous stirring. After that, concentrated ammonia aqueous solution (0.6 mL), formaldehyde (0.23 mL), and tetraethylorthosilicate (TEOS, 0.8 mL, 3.6 mmol) were added into the solution in sequence. The reaction mixture was then stirred at 40°C for 10 h followed by heating to 80°C and standing in static for 24 h. After filtration and washing with water, the as-prepared sample was brown-reddish powders and denoted as **S1**. Thereafter, sample **S1** was annealed at 350°C for 2.0 h and then at 1,400°C for 4.0 h with a heating rate of 2.0°C· min^−1^ in Ar atmosphere to gain the as-synthesized SiC product, denoted as **S2**. After the sample was cooled down to room temperature naturally, it was immersed into10 wt% HF aqueous solution for 24 h at room temperature to completely remove the resident SiO_2_, and finally washed with deionized water for three times.

### The electrochemical measurements

Electrochemical tests were performed using CR2016 coin-type cells assembled in an Ar-filled glove box. For preparation of the working electrode, **S2** (80 mg), acetylene black (AB, 10 mg) and binder polyvinylidene fluoride (10 mg, dissolved in N-methylpyrrolidone) were mixed together. The resultant slurry was then uniformly coated on a Cu foil current collector and dried overnight under vacuum. Electrochemical cells were assembled using **S2** electrode as the cathode, metallic lithium foil as the anode, Celgard 2325 porous film as the separator, and 1.0 M LiPF_6_ solution (dissolved in a mixed solvent of ethylene carbonate and diethyl carbonate, 1:1 by volume) as the electrolyte. The cells were charged and discharged between 3.0 and 0.01 V at room temperature.

## Results and discussion

**Figure 1:**
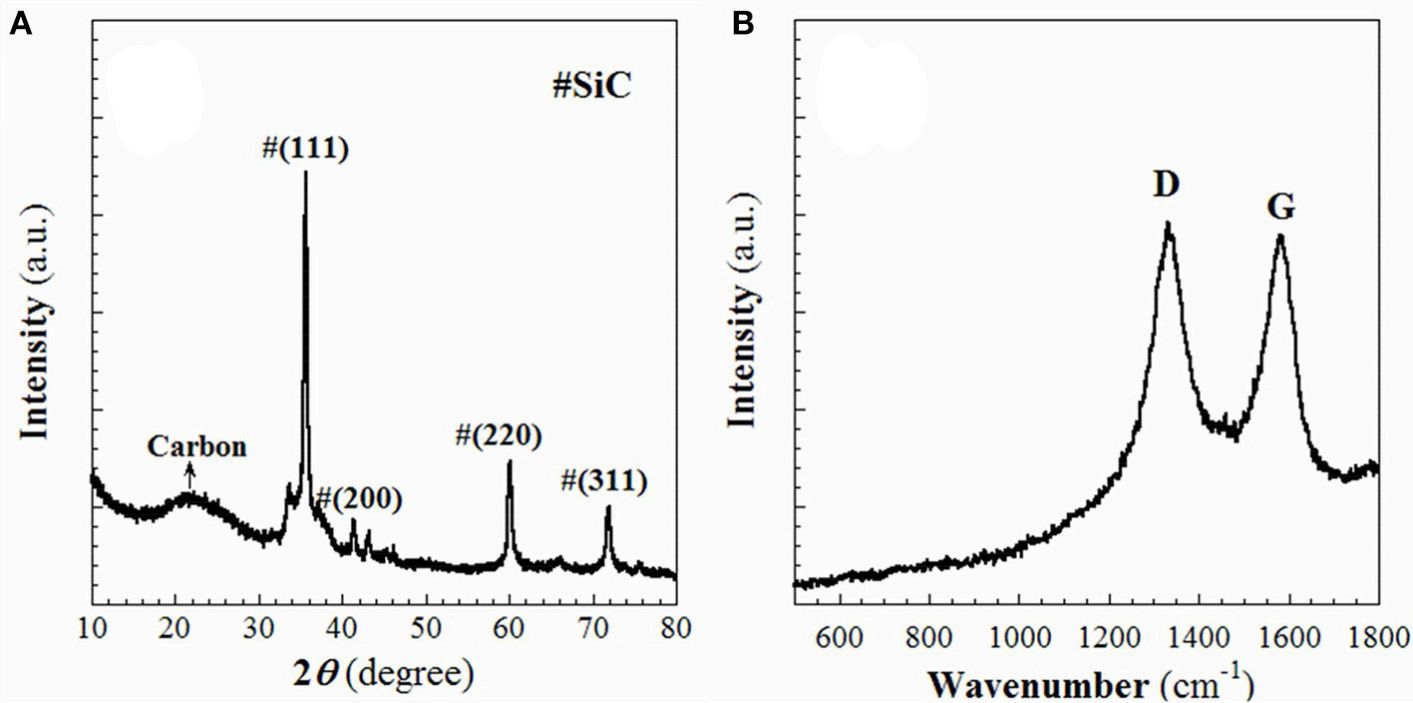
**(A)** WAXRD pattern and **(B)** Raman spectrum of the sample **S2**.

**Figure 2:**
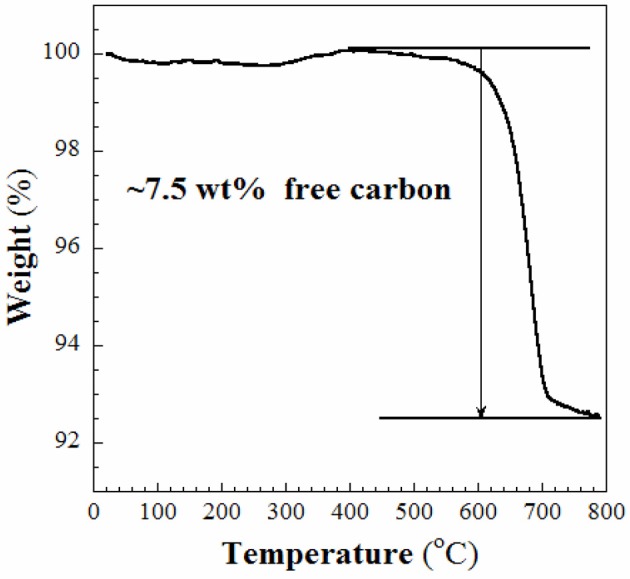
TGA curve of the sample **S2**.

In this work, the RF resin-silica composite **(S1)** was used as the source to fabricate aimed product-SiC as mentioned in the experimental section. The template and RF resin in **S1** was firstly converted to C at lower temperature (~600°C) when heated in Ar. When the temperature rose up to 1,400°C, the silica in **S1** reacts with the C to produce SiC. The obtained sample **S2** was firstly characterized using WAXRD and Raman spectroscopy. As shown in Figure 1, in the WAXRD pattern (Figure 1A), peaks displayed at 2θ = 35.6, 41.4, 60.1, and 71.9° are indexed to (111), (200), (220), and (311) planes of cubic β-SiC phase (JCPDS: 29-1129). At the same time, a broad peak centered at 2θ = 22.9° is ascribed to the (200) plane of graphite. There are not any diffraction lines that could be assigned to SiO_2_, which suggests that the SiO_2_ compound has been safely removed. In the Raman spectrum (Figure 1B), the D and G bands appear at 1,331 and 1,581 cm^−1^, respectively. The value of *I*_G_/*I*_D_ is 0.95, indicating that the carbon in sample **S2** is predominantly amorphous. Due to the relatively low intensities of SiC Raman shifts compared to those of C, it is difficult to define the SiC Raman peaks in Figure 1B. TGA analysis, shown in Figure 2, discloses that ~7.5 wt% of free carbon presents in the sample **S2**. These results show that **S1** has been successfully converted to crystalline SiC with small amount of residual carbon.

**Figure 3:**
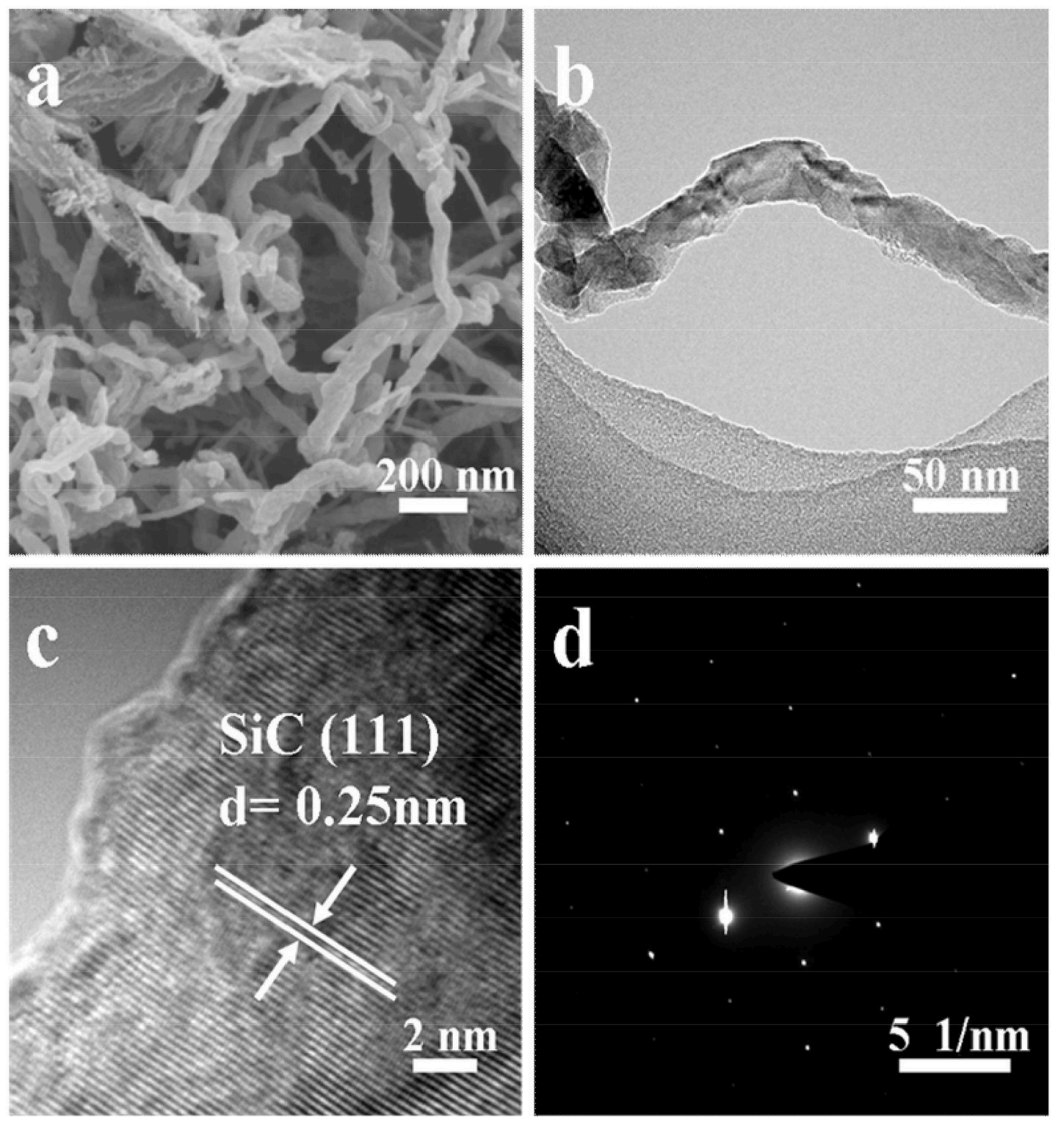
**(a)** FE-SEM, **(b)** TEM and **(c)** HR-TEM images, and **(d)** SAED pattern of sample **S2**.

**Figure 4:**
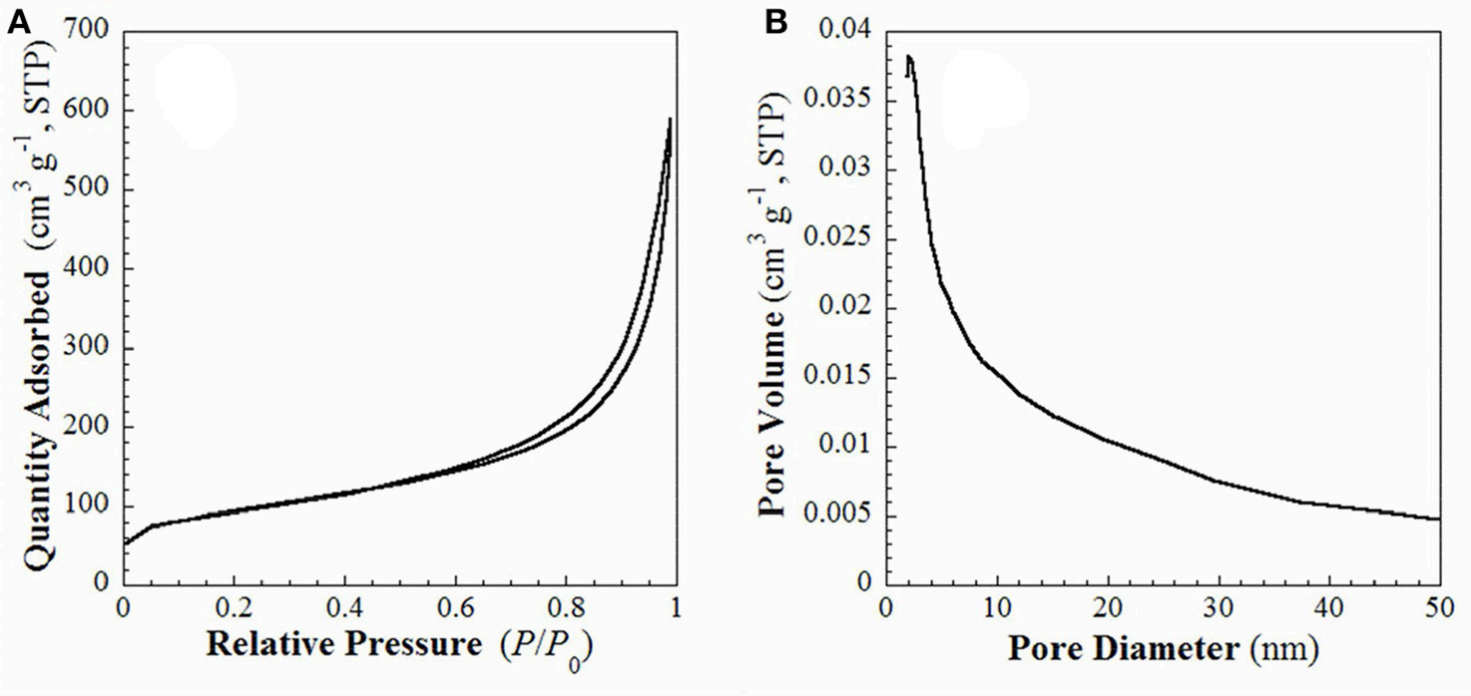
**(A)** N_2_ adsorption-desorption isotherms and **(B)** BJH pore size distribution plot calculated from the adsorption branch of sample **S2**.

FE-SEM and TEM images of the samples **S1** and **S2** are shown in Figure S2 and Figure 3. Sample **S1** are right-handed helical nanofibers with width, wall thickness and helical pitch of 80–150, 15–30, and 800–1,200 nm, respectively. The lengths of the nanofibers are several microns as observed in Figure S2. After pyrolysis, the sample **S2** exhibits twisted fiber-like morphology, as shown in Figures 3a,b. The average length and diameter of the nanofibers are several microns and 30–50 nm. In the HRTEM image (Figure 3c), the interplanar distance is 0.25 nm, which is consistent with the lattice fringes of cubic β-SiC (111) face. The selected area electron diffraction (SAED) pattern (Figure 3d) implies that the obtained SiC is highly crystalline. These observations are consistent with the results of WAXRD analysis in Figure 1. The N_2_ adsorption-desorption isotherms and the BJH pore size distribution plots calculated from the adsorption branch for the sample **S2** are shown in Figure 4. It shows type-IV isotherms with H3-hysteresis loop, which are the characteristic of microporous and mesoporous materials. These micro- and mesopores are produced mainly due to the breakup of C-C bonds and formation of crystalline SiC during the pyrolysis process as well as the dissolution of SiO_2_ compound during the HF solution treatment. The BET specific surface area and BJH adsorption pore volume are 326.4 m^2^·g^−1^ and 0.86 cm^3^·g^−1^, respectively.

**Figure 5:**
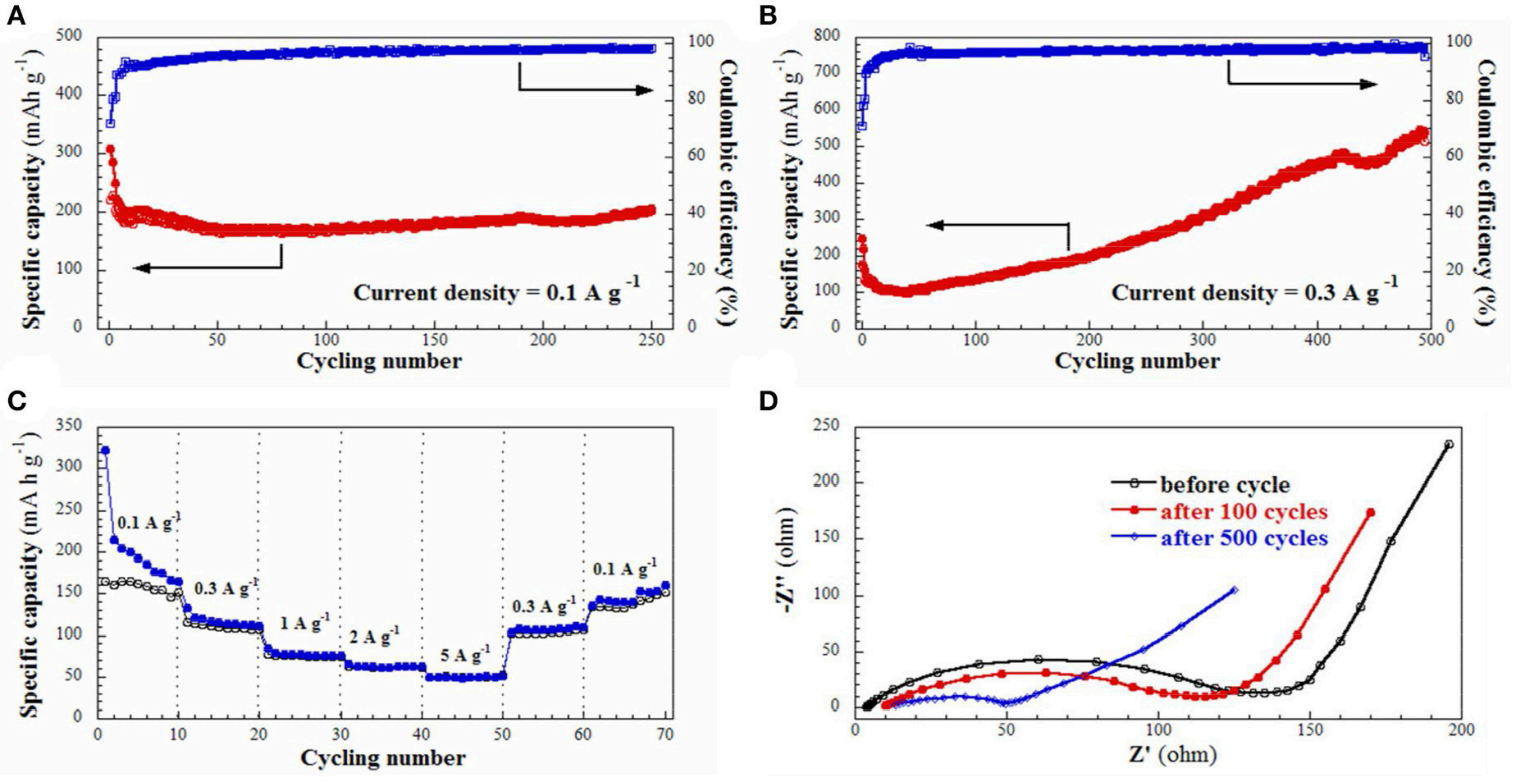
**(A,B)** Cycling behavior of **S2** electrodeat current density of 0.1 A g^−1^ and 0.3 A g^−1^; **(C)** Rate performance of **S2** electrode at various current densities from 0.1 A g^−1^ to 5 A g^−1^; **(D)** Nyquist plot for **S2** electrode in the frequency range from 100 KHz to 10 MHz before and after cycling.

Cycling performance of the **S2** electrode was evaluated, as shown in Figure 5A. The first discharge and charge capacities are 309.3 and 221.9 mAh g^−1^, respectively, giving an initial Coulombic efficiency of 71.7%, however, which quickly increases and keeps at above 97% in the following cycles. The discharge capacity quickly decreases to 195.4 mAh g^−1^ in the 8th cycle, thereafter stabilizes at about 170 mAh g^−1^ and rises slowly to 205.4 mAh g^−1^ in the 250th cycle. Apparently, the relatively low capacity of the **S2** electrode is primarily attributed to its low carbon content and the electrochemically inertial nature of SiC. Moreover, the cycling performance of the **S2** electrode tested at a higher current density of 0.3 A g^−1^ is also shown in Figure 5B. In the initial 40 cycles, the discharge capacity decreases from 247.9 to 101.8 mAh g^−1^. After that, it starts to increase gradually, and reversible capacities of 254.5 mAh g^−1^ at the 250th cycle and 540.1 mAh g^−1^ at the 500th cycle are obtained. It seems that high current density will accelerate the activation of SiC electrode. Clearly, the SiC electrode exhibits higher capacity than the commercial graphite electrode (~370 mAh g^−1^) and also better cycling stability than the common Si electrode (Zhang et al., 2014).

**Figure 6:**
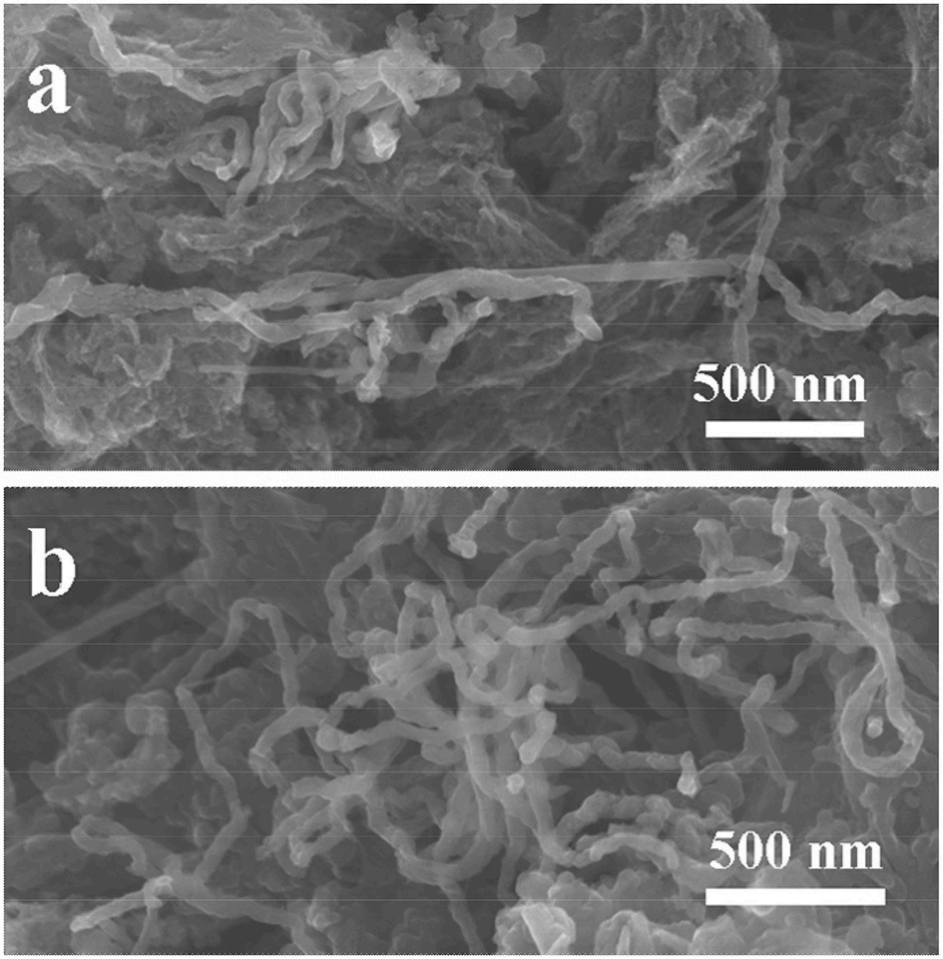
FE-SEM images of the **S2** electrode after cycling: **(a)** 250 cycles at 0.1 A g^−1^; **(b)** 500 cycles at 0.3 A g^−1^.

It's interesting to observe that the capacity increases after long cycles at the current density of 0.3 A g^−1^, as displayed in Figure 5B. We firstly reported similar capacity ascending curve for the SiC/C composite (containing 17 wt% carbon, at the current density of 0.1 A g^−1^), which was attributed to a gradually activation of electrode in the lithiation/delithiation process similar to some metal oxides (Cheng et al., 2013; Wang C. et al., 2015; Sun et al., 2016; Li J. et al., 2017; Shao et al., 2017a). Zhang and Xu also reported the capacity increasing phenomenon for the nanocrystalline SiC from the second cycle. They proposed the reduction of Si^4+^ to Si^0^ and the formation of Li-C and Li-Si alloys in the electrode (Zhang and Xu, 2014). This is more rather correct explanation, because a high current density will fasten the reaction of Li with SiC nanomaterials to form Li-Si and Li-C alloys. Consequently, after tens times of cycling, the SiC electrode will slowly convert to Si/C nanostructure composite, because the re-formation of SiC from Si/C composite needs at rather high temperature. In this case, previously electrochemical inert SiC electrode gradually transfer to electrochemically active Si/C composite electrode, which will interpret why the electrode capacity keeps growing at 0.3 A g^−1^ current density. In order to prove our hypothesis, the **S2** electrode was cycled at 5 A g^−1^ as shown in Figure S3. Its capacity decreases to about 76 mAh g^−1^ at the 16th cycle, and then gradually increases to 128 mAh g^−1^ at the 40th cycle. It is found that the capacity enhancement is much earlier and faster and the electrodes cycled at 0.1 and 0.3 A g^−1^. It is very interesting to find that after SiC partially transfer to Si/C composite, it still maintains original nanofiber structure. This may explain why the SiC electrode could possess a rather good cycling stability. FE-SEM images of the **S2** electrode after cycling test were then taken and showed in Figure 6. The nanofibers keep original morphology, and no changes in lengths or diameters are observed. Figure S4 shows the XRD patent of the electrode after cycling at 5 A g^−1^ for 40 times. It could be seen that the bump for C species slightly increases and the diffractions for the SiC decreases, which indicates the change of component in the SiC/C composite. Out of our expectation, the signals for Si were not observed in the patent, which may because that the Si decomposed from SiC is not crystalline but amorphous.

Unlike Si-based electrodes, which suffer from huge volume changes during the lithiation/delithiation process, resulting in the pulverization of Si nanostructure and consequently shortening the cycling properties of the batteries. SiC nanostructures have rather high hardness, the partial decomposition of SiC into to Si and C composites would not alter the base structure of SiC, meanwhile, the decomposed Si nanoclusters could well distribute into the C matrix. This may explain why after long-time cycling the Si/C composite conversed from SiC/C still preserves nanofiber structure. The Raman spectra of **S2** electrode after cycling have been measured and are showed in Figure S5. The values of *I*_G_/*I*_D_ are 0.86 at the current density of 0.1 A g^−1^ after 250 cycles and 0.94 at the current density of 0.3 A g^−1^ after 500 cycles, respectively. Compared with the sample before cycling (the *I*_G_/*I*_D_ value is 0.95), the graphitization change is small. It is reasonable to suggest that the decomposed C from SiC is also amorphous, because under the electrochemical reactions it is not easy to form crystalline carbon nanostructure during lithiation/delithiation.

Compared with our previously reported results, the C content in the SiC/C composites exhibited great effect on the capacity, as listed in Table 1. At low current density (0.1 A g^−1^), pure carbon shows the biggest capacity value, and the small amount of SiC will lower the capacity because of its electrochemical inertness. When SiC is the major moiety, the capacity decreases with the content of C after long cycles. However, at higher current density (over 0.3 A g^−1^), the SiC/C composites show higher capacity than pure carbon after long cycles, because high current density will accelerate the activation of SiC electrode. The SiC transforms into Si/C composite keeping original SiC nanostructure (Han et al., 2011). The theoretical capacity of Si is much higher than that of C (Ren et al., 2013, 2014; Sun et al., 2015; Li H. et al., 2017), therefore, SiC/C composites would show both higher capacity than C materials and superior stability than Si materials. The relative lower capacity values obtained in current work is due to the lower original C content, which may slowly increase with the cycling number.

**Table 1:** Electrochemical performance of SiC-C nanocomposites toward LIBs anode materials.

**Sample**	**C content (wt%)**	**Current density (A g^−1^)**	**Reversible capacity**	**References**
C nanorods	100	0.1	696.7 mAh g^−1^ after 400 cycles	Hu et al., 2018
		0.37	289.3 mAh g^−1^ after 400 cycles	
C/SiC nanorods	90.4	0.1	457.9 mAh g^−1^ after 400 cycles	Hu et al., 2018
		0.37	314.6 mAh g^−1^ after 400 cycles	
SiC/C nanotubes	31	0.1	527 mAh g^−1^ after 250 cycles	Shao et al., 2017a
		0.3	600 mAh g^−1^ after 500 cycles	
SiC/C nanorods	17	0.25C^*^	283.6 mAhg^−1^ after 250 cycles^**^	Wang C. et al., 2015
SiC/C nanofibers	7.5	0.1	205.4 mAh g^−1^ after 250 cycles	Present work
		0.3	504.1 mAh g^−1^ after 500 cycles	

* *1C = 0.37 A g^−1^*;

** *the data wasn't marked in Timmons et al. (2007)*.

The rating capability of the **S2** electrode was evaluated at various current densities from 0.1 to 5 A g^−1^, as shown in Figure 5C. Reversible capacities of 165.1, 111.7, 76.2, 62.9, 51.5, 110.1, and 160.1 mAh g^−1^ are obtained, respectively, indicating excellent rating capability. To study the conductivity of the **S2** electrode, the electrochemical impedance spectra (EIS) were also collected and shown in Figure 5D, which illustrates an impedance of 180 Ω before cycling, 120 Ω after 100 cycles, and 50 Ω after 500 cycles. The rapidly decreased impedance implies that the SiC electrode after cycling also possesses a good electrical conductivity and a rapid charge-transfer reaction for Li ion insertion and extraction. This result is consistent with our above proposal that SiC would gradually decompose into Si and C during electrochemical Li insertion/extraction.

## Conclusions

Novel SiC nanofibers derived from resorcinol-formaldehyde resin/silica composites were designed and synthesized successfully. When used as anode materials for LIBs, they exhibited superior cycling stability, good rating capability and low impedance. A special capacity increasing phenomenon was observed after long cycles, which was ascribed to the partial decomposition of SiC nanostructure into Si/C composites during the lithiation/delithiation process. In addition, the effect of C content in the SiC/C composite was compared and discussed. The results in this work disclose that the nano-sized SiC materials are promising anode candidate for long-life LIBs due to their high nanostructure stability.

## Author contributions

All authors listed have made a substantial, direct and intellectual contribution to the work, and approved it for publication.

### Conflict of interest statement

The authors declare that the research was conducted in the absence of any commercial or financial relationships that could be construed as a potential conflict of interest.
