# Supplementary material for: SiC Nanofibers as Long-Life Lithium-Ion Battery Anode Materials
Source: Front Chem. 2018 May 14;6:166. doi: 10.3389/fchem.2018.00166 (PMC5960690; doi:10.3389/fchem.2018.00166)
Supplement: Supplementary file 1 [file Data_Sheet_1.DOC]

**Supporting Information**

**SiC nanofibers as long-life lithium-ion battery anode materials**

Xuejiao Sun^1§^, Changzhen Shao2[[1]](#footnote-2)§, , Feng Zhang^2^, Yi Li*^, 2^, Qi-Hui Wu*^, 1^, Yonggang Yang^2^

^1^ Department of Materials Chemistry, School of Chemical Engineering and Materials Science, Quanzhou Normal University, Quanzhou 362000, P.R. China

^2^ Jiangsu Key Laboratory of Advanced Functional Polymer Designand Application, Department of Polymer Science and Engineering, College of Chemistry, Chemical Engineering and Materials Science, Soochow University, Suzhou 215123, P.R. China.


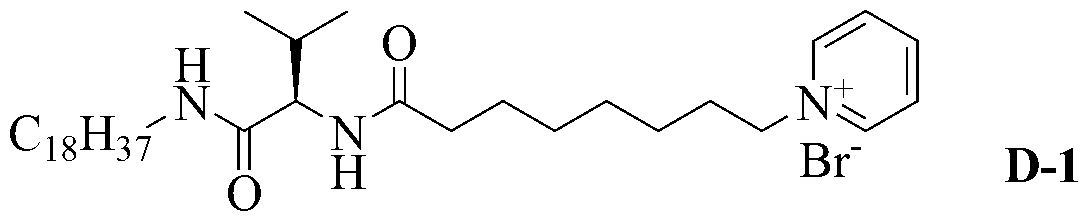


Figure S1. Molecular structure of the gelator D-1.


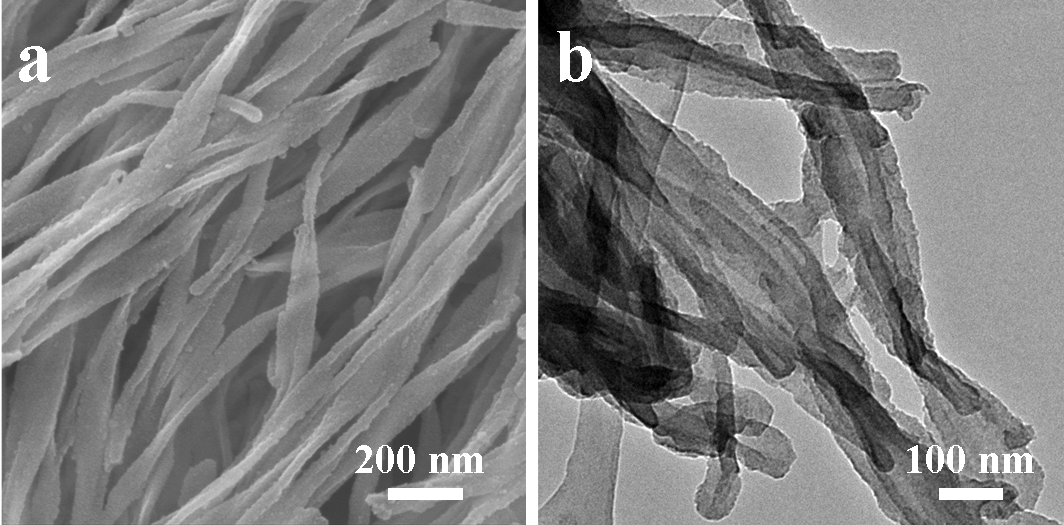


Figure S2. (a) FE-SEM and (b) TEM images of sample **S1**.

Figure S3 The XRD patents of SiC/C electrode before and after 48 cycles at current density of 5 A/g.

Figure S4 The cycling performance of SiC/C electrode at a current density of 5 A/g.


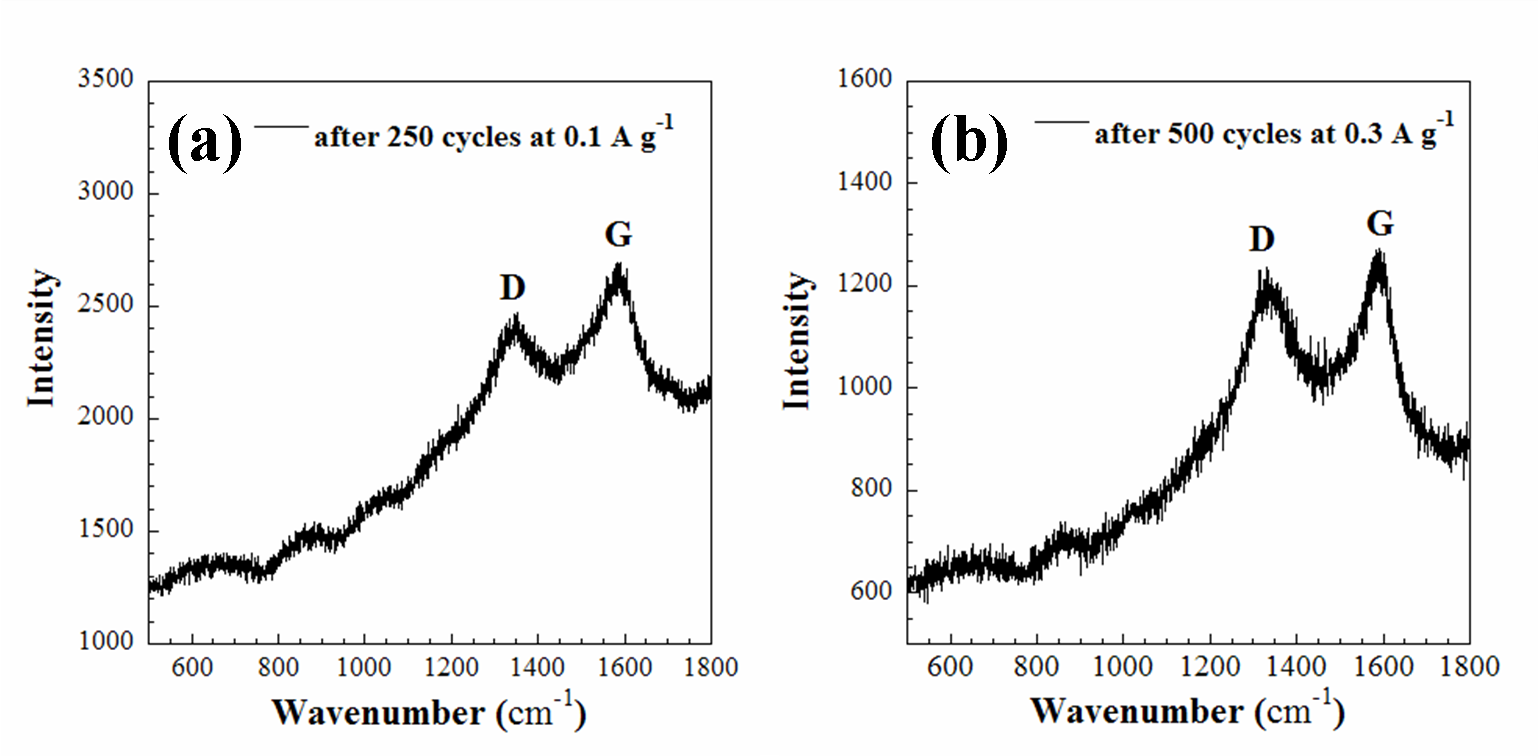


Figure S5. Raman spectra of the S2 electrode after cycling: 250 cycles at 0.1 A g^-1^; (b) 500 cycles at 0.3 A g^-1^.

1. ^§^ Equal contribution [↑](#footnote-ref-2)
